# Psychometric properties of the Arabic version of the cardiac depression scale tested on Jordanian patients with cardiovascular diseases

**DOI:** 10.1186/s12888-020-02651-8

**Published:** 2020-05-19

**Authors:** Ibtisam M. Al-Zaru, Audai A. Hayajneh, Tariq Al-Dwaikat

**Affiliations:** 1 Adult Health Nursing Department, Faculty of Nursing, Jordan University of Science and Technology, P.O. Box: 3030, Irbid, 22110 Jordan; 2 Community and Psychiatric Health Nursing Department, Faculty of Nursing, Jordan University of Science and Technology, P.O. Box: 3030, Irbid, 22110 Jordan

**Keywords:** Cardiac depression scale, Cardiac, Psychometric properties, Arabic, Jordan

## Abstract

**Background:**

The Cardiac Depression Scale (CDS) is an instrument to screen for depression, specifically in patients with cardiovascular diseases (CVD). The purpose of this study was to evaluate the psychometric properties of the Arabic version of the CDS in the Jordanian population.

**Method:**

A cross-sectional design was used for 304 participants diagnosed with CVD. An exploratory factor analysis (EFA) was performed to explore the underlying structure of the new Arabic version of the CDS tool.

**Results:**

Cronbach’s alpha for the total scale was 0.87. An EFA suggested a two-factor solution. The first factor has 18 items, measuring “My tolerance” of CVD, and the second factor has seven items, measuring “My activities” in the context of CVD. Based on the EFA simple structure, one item was removed due to its low factor loading (< 0.3). A confirmatory factor analysis (CFA) supported a two-factor model with the root mean square error of approximation (RMSEA = 0.06), comparative fit index (CFI = 0.856), and Tucker-Lewis index (TLI = 0.83) indicating acceptable fit. The Cronbach’s alpha values for the first and second factors were 0.86 and 0.84, respectively.

**Conclusion:**

The Arabic version of the CDS is a reliable and valid instrument to screen for depression among Jordanian patients with CVD.

## Background

Cardiovascular diseases (CVD) are the most common non-communicable diseases globally and were responsible for an estimated 17.8 million deaths in 2017. More than three quarters of CVD deaths were in low-income and middle-income countries [1, 2]. CVD is the leading cause of death among Jordanian adults, accounting for about 37% of all deaths in Jordan [3]. Depressive symptomatology is common among patients with CVD, and affects approximately 20 to 40% of all patients [4]. The coexistence of CVD and depression appears to be bidirectional [5, 6].

Depression was found to be associated with an increase in the occurrence of cardiovascular events, worsening health-related quality of life, and increased mortality rate due to various pathophysiological mechanisms [4, 7]. Depression is observed in more than 50% of patients diagnosed with CVD, but is usually alleviated with appropriate management and support. If depressive symptoms are left unaddressed in CVD patients, they can develop into a major depressive disorder [7]. The health-related quality of life of patients with coronary artery diseases (CAD) is significantly affected by coexisting depressive symptomatology [8, 9]. The incidence and prevalence of depression in patients with CVD in Jordan is unknown as there is no screening or tracking for depression currently. Therefore, early identification of depression is essential to provide appropriate intervention for these patients, so that they can be successfully rehabilitated to their routine life. Researchers have examined the effect of intervention on depression in patients with CAD [10]. For instance, social support plays a major role in managing both cardiovascular diseases and depression at the same time. Greater perceived social support can lead to an improvement in depressive symptoms, which in turn can improve the patient’s overall quality of life [10]. The perception of social support has been linked to the prognosis of CAD and the associated mortality [11]. Low functional social support can be a risk factor for developing CAD and can impact CAD-related mortality [11]. In addition, the absence of social support can lead to a poor prognosis and adverse outcomes in patients diagnosed with CVD [12]. Furthermore, low social support in those with cardiovascular events are major predictors of depression in patients with CVD [13]. Thus, it is crucial to assess depression in patients with CVD.

Researchers use various self-report questionnaires to assess depression in patients with CVD. The most frequently used questionnaires include: the Patient Health Questionnaire (PHQ) [14], Beck’s Depression Inventory (BDI) [15], the Hospital Anxiety Depression Scale (HADS) [16], the Center for Epidemiologic Studies Depression Scale-10 (CES-D) [17], and the Cardiac Depression Scale (CDS) [18]. Among these instruments, only the CDS is designed specifically for screening depression in patients with CVD. The CDS is specifically beneficial in cardiac settings compared to the BDI.

### Description of the instrument

The CDS was developed and validated by Hare and Davis (1996), and comprises 26 items clustered in seven subscales. The subscales are sleep, uncertainty, mood, hopelessness, inactivity, anhedonia, and cognition. The items are rated on a 7-point Likert scale, with the seven items reverse-coded. Higher total scores are indicative of more severe levels of depression [18]. The CDS demonstrated high consistency reliability (α = 0.90) and excellent criterion validity compared to the BDI [18].

The CDS was validated in studies that recruited patients with CVD. For example, Kiropoulos and colleagues [19] tested the CDS, the BDI and the State Trait Anxiety Inventory (STAI) on a group of 152 patients with coronary heart disease (CHD). Their results supported the dimensionality of the original scale, as well as the concurrent validity of the CDS compared to the BDI; the STAI was also supported. In another British study on patients with CHD, the concurrent validity of CDS was established compared to the HADS and the BDI, in which Cronbach’s αlpha for CDS was found to be 0.93 in a sample of 395 patients [20].

A systematic review of the studies that evaluated the psychometric properties of the CDS concluded that the CDS was a psychometrically reliable measure of depression in patients with CHD [21]. Chavez and colleagues found that the CDS was a highly reliable and valid instrument with very good sensitivity in diagnosing depression in patients with CVD. To date, no other measure has been designed specifically to assess depression in patients with CHD [21]. The CDS was translated into Persian [22] and Chinese [23]. Both studies showed satisfactory internal consistency reliabilities. Wang and colleagues’ study [23] had a Cronbach’s α of 0.91 in a sample of 200 participants, and Gholizadeh and colleagues’ study [22] had a Cronbach’s αlpha of 0.88 in a sample of 261 participants. These psychometric properties of the CDS reported for the Chinese and the Iranian cultures support the feasibility of using the CDS as a screening tool for depression in patients with CVD in the Jordanian population as well.

The CDS was also translated and validated in the Arabic language [24]. Papasavvas and colleagues assessed the psychometric properties of the Arabic version of the CDS in a sample of 260 participants from one clinical setting and confirmed the feasibility of using the CDS with Arabic speaking patients. Although, the participants in Papasavvas and colleagues’ study were from 18 different Arab countries, the majority (54%) were from two countries: Egypt and Qatar. The feasibility of the CDS in the cultural context of the Jordanian population has not been assessed yet.

The research team translated the CDS into Arabic (Jordanian) to screen Jordanian patients with CAD. This approach was used because the previous Arabic version of the CDS was not culturally applicable and adequate for the Jordanian population. Although several countries use Arabic as their national language, there are several differences in the words and the meanings of words (i.e., different accents). Therefore, this translation (Arabic-Jordanian) was used in two studies to screen Jordanian patients with CAD for depression. The purpose of this secondary data analysis was to evaluate the psychometric properties of the Arabic version of the CDS in a sample of Jordanian patients diagnosed with CAD.

## Methods

### Design and sample

A secondary data analysis was performed on data collected from two previous studies. The Arabic-Jordanian version of the newly translated CDS tool [25] was used in this study, which is different from the previous translation used in a Lebanese study [24]. The first study [25] examined the presence of depressive symptomology among 174 non-hospitalized Jordanian patients diagnosed with CAD. Their health-related factors and depression were investigated, and predictors of depression were identified using the Arabic version of the CDS. The second study [26] involved 130 Jordanian participants diagnosed with CAD who completed the same Arabic version of the CDS. Cross-sectional data collected from both these studies were combined to obtain the final number of participants for this study, which was 304 participants. These participants were from the northern part of Jordan, where Arabic is the primary language. All the participants in both studies were above 18 years old and were recruited using convenience sampling from the outpatient unit of a large referral hospital through face-to-face interviews.

### Cardiac depression scale (CDS): forward translation and Back-translation

The CDS is a 26-item self-report questionnaire that uses a 7-point Likert scale [1–7] to measure depression in patients with CVD. This scale has three dimensions, and was developed in English and validated in an Australian population. They found that the reliability coefficient was 0.9 and correlated with BDI (r = 0.73) [18]. This tool was translated into Persian and Arabic (Lebanese) versions. However, the Arabic spoken in Jordan is different from that of Lebanon; therefore, it was necessary to translate it into Jordanian Arabic to gather accurate data from Jordanian patients with CVD.

Thus, the CDS was translated into Jordanian Arabic after obtaining approval from the developers of the original CDS [18]. Two bilingual experts translated the original CDS tool from English into Arabic, after which two other professional experts proficient in Arabic and English were asked to perform back-translation into English. Thereafter, both versions were checked for equivalence. Discrepancies between the Arabic and English translations were resolved by the researchers. All the items were checked and approved for face validity and introduced to the participants. None of the items were deleted for any reason.

### Procedures

The two studies were approved by the Institutional Review Boards of Jordan University of Science and Technology (IRB # 216–274) and participants signed a written consent form. Participants were diagnosed as patients with CAD, who were receiving follow-up treatment at the cardiac outpatient unit. They were asked to complete the new Arabic (Jordanian) version of the CDS self-report questionnaire through face-to-face interviews to make sure that all participants understood the questions.

### Data analysis

All analyses were performed using the JASP version 0.8.6 statistical software [27]. First the assumptions of normality, linearity, homogeneity, and homoscedasticity were checked. Outliers were removed from the analyses prior to finalizing a sample size of 304 participants. Reliability and validity analyses of the CDS were performed. The internal consistency reliability analysis (Cronbach’s α) was checked for the total scale and the two resultant factors. An exploratory factor analysis (EFA) was performed to explore the underlying factor structure of the new Arabic version of the CDS in order to examine the quality of the individual items to reflect depression. Preacher and MacCallum’s guidelines [28] were used to conduct the EFA analyses. EFA is usually used as a first step in validating a translated instrument in a new population—in this study, the Jordanian population [29]. A confirmatory factor analysis (CFA) was performed using maximum likelihood estimation to confirm the EFA structure and check for model fitness. In order to investigate the models’ goodness of fit, a number of statistics were used: overall χ2, root mean square error of approximation (RMSEA) [30], comparative fit index (CFI), and the Tucker-Lewis index (TLI) [31].

## Results

Three hundred and four participants were asked to answer a self-reported questionnaire on the CDS through interviews. The mean age of the participants was 57.59 (SD: 10.56). Just over half of the participants were female (51.3%). Approximately, 57.2% of participants were smokers, and 37% of participants had acquired secondary education or less.

### Reliability

An internal reliability analysis (Cronbach’s alpha) was used to explore the internal consistency of the Arabic version of the CDS using JASP. Assumptions of multivariate were checked (normality, linearity, homogeneity, and homoscedasticity) and no violations were found. A Cronbach’s alpha above 0.7 is usually considered as acceptable reliability of a given tool [32]. The total scale reliability of the Arabic version of the CDS was 0.87, which is very good, and shown in Table 1. Results did not suggest the need for removal of any items (Table 2). The exploratory factor analysis is discussed in a separate section below; results of this analysis suggested the use of two factors model to establish the simple structure of the scale. The two reliability values of factors one and two were 0.86 and 0.84, respectively, which are considered good, as shown in Table 1.

**Table 1:** Scale Reliability Statistics for Total, Factor One and Factor Two

	**mean**	**SD**	**Cronbach’s α**	**95.0% Confidence Interval**	
				**Lower**	**Upper**
Total	3.756	0.573	0.867	0.844	0.887
Factor One	3.683	0.642	0.857	0.833	0.879
Factor Two	4.025	0.255	0.843	0.814	0.868

**Table 2:** Item Reliability Statistics

	**If item dropped**
	**Cronbach’s α**
I have dropped many of my interests and activities	0.864
My concentration is as good as it ever was	0.863
I can’t be bothered doing anything much	0.860
I get pleasure from life at present	0.862
I am concerned about the uncertainty of my health	0.859
I may not recover properly	0.866
My sleep is restless and disturbed	0.864
I am not the person I used to be	0.856
I feel like I’m living on borrowed time	0.861
Dying is the best solution for me	0.864
I feel in good spirits	0.860
The possibility of sudden death worries me	0.863
There is only misery in the future for me	0.860
My mind is as fast and alert as always	0.861
I get hardly anything done	0.860
My problems are not yet over	0.861
Things which I regret about my life are bothering me	0.863
I gain just as much pleasure from my leisure activities as I used to	0.863
My memory is as good as it always was	0.864
I become tearful more easily than before	0.867
I seem to get more easily irritated by others than before	0.858
I feel independent and in control of my life	0.863
I lose my temper more easily nowadays	0.862
I feel frustrated	0.856
I am concerned about my capacity for sexual activity	0.865

### Validity

**Fig. 1:**
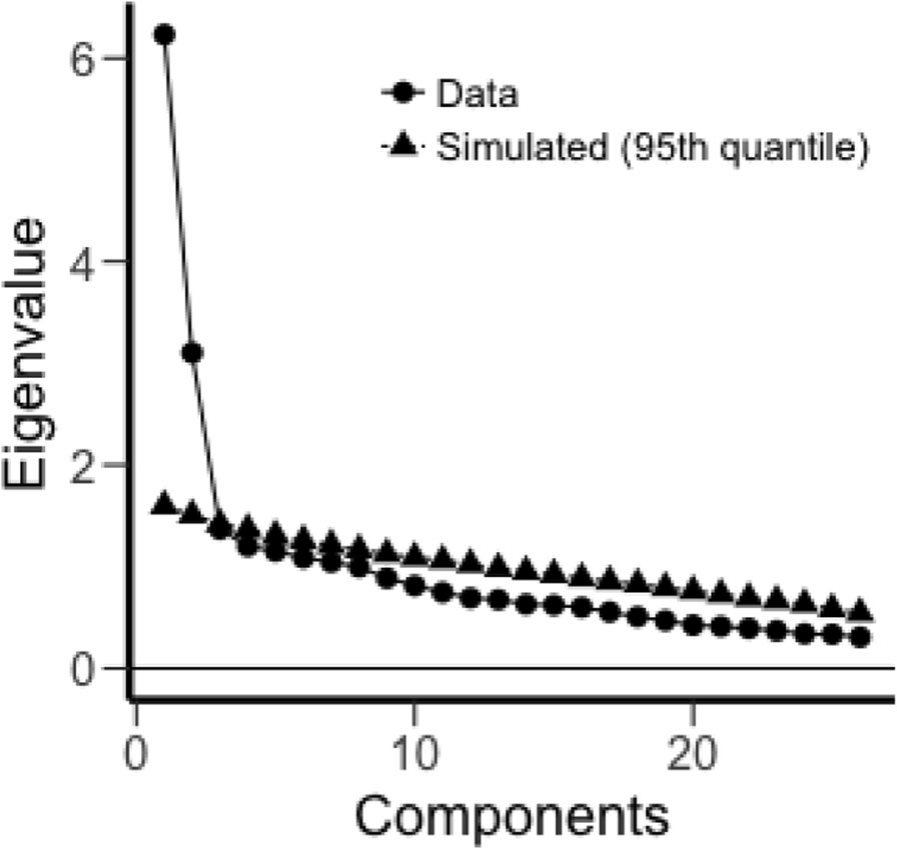
Scree plot of the total scale

An EFA was used to explore the underlying factor structure of the Arabic (Jordanian) version of the CDS according to Preacher and MacCallum’s (2003) guidelines. A parallel analysis suggested five factors, and the Kaiser method suggested ten factors. However, a scree plot (Fig. 1.) and the underlying theoretical basis recommended two factors. Although a previous study suggested three factors for the Arabic version of the CDS [24], the suggestion of the scree plot and the underlying theoretical basis was followed in this study. Thus, two factors were tested for the Arabic (Jordanian) version of the CDS. A maximum likelihood examination with a promax rotation was used because of the possibility of a correlation between the factors. Twenty-six [26] items were tested using the EFA analysis. Promax rotation was used and 0.30 was set as the cut-off point for factor loading. One item did not load above 0.30 (“I wake up in the early hours of the morning and cannot get back to sleep”). This item was removed to re-run the EFA analysis. Then, the simple structure of a two-factor model was achieved with 25 items, as shown in Table 3. Additional fit indices were analyzed to check the fitness of this model. One of tests for errors (RMSEA) was acceptable (0.062) while another (TLI) was not acceptable (0.838). Chi-squared test was significant (526.062, *p* < 0.001).

**Table 3:** Component Loadings of the Two-factors Model of the Cardiac Depression scale

**Item**	**Factor 1**	**Factor 2**	**Uniqueness**
My concentration is as good as it ever was	0.017	**0.576**	0.661
I feel independent and in control of my life	−0.010	**0.715**	0.493
I become tearful more easily than before	**0.469**	−0.208	0.802
I seem to get more easily irritated by others than before	**0.677**	−0.074	0.569
I have dropped many of my interests and activities	**0.488**	−0.068	0.779
I feel like I’m living on borrowed time	**0.584**	−0.060	0.679
I can’t be bothered doing anything much	**0.447**	0.157	0.729
Dying is the best solution for me	**0.410**	0.022	0.826
I feel frustrated	**0.700**	0.040	0.490
I get hardly anything done	**0.517**	0.042	0.716
There is only misery in the future for me	**0.504**	0.082	0.712
I may not recover properly	**0.325**	−0.002	0.895
I gain just as much pleasure from my leisure activities as I used to	−0.000	**0.560**	0.687
I am not the person I used to be	**0.675**	0.059	0.515
My memory is as good as it always was	−0.050	**0.652**	0.594
My mind is as fast and alert as always	0.036	**0.661**	0.546
I get pleasure from life at present	−0.024	**0.658**	0.577
My problems are not yet over	**0.484**	0.078	0.734
Things which I regret about my life are bothering me	**0.463**	0.020	0.779
I am concerned about my capacity for sexual activity	**0.325**	0.076	0.872
My sleep is restless and disturbed	**0.473**	−0.046	0.789
I feel in good spirits	0.000	**0.793**	0.371
The possibility of sudden death worries me	**0.428**	0.050	0.800
I lose my temper more easily nowadays	**0.529**	−0.065	0.739
I am concerned about the uncertainty of my health	**0.593**	0.028	0.636

As shown in Table 3, factor one included 18 items that measured “My tolerance” of CDS with questions such as “I become tearful more easily than before,” “I seem to get more easily irritated by others than before,” and “I have dropped many of my interests and activities.” These questions are shown in Appendix A for the questionnaires. Factor two included seven items that measured “My activities” in the context of CDS, with questions such as “My concentration is as good as it ever was.” The reliability of the CDS, factor one, and factor two were good with scores of 0.867, 0.857, and 0.843, respectively. The mean scores for each factor were: factor one *M* = 3.683 (*SD* = 0.642), and factor two *M* = 4.025 (*SD* = 0.255).

**Fig. 2:**
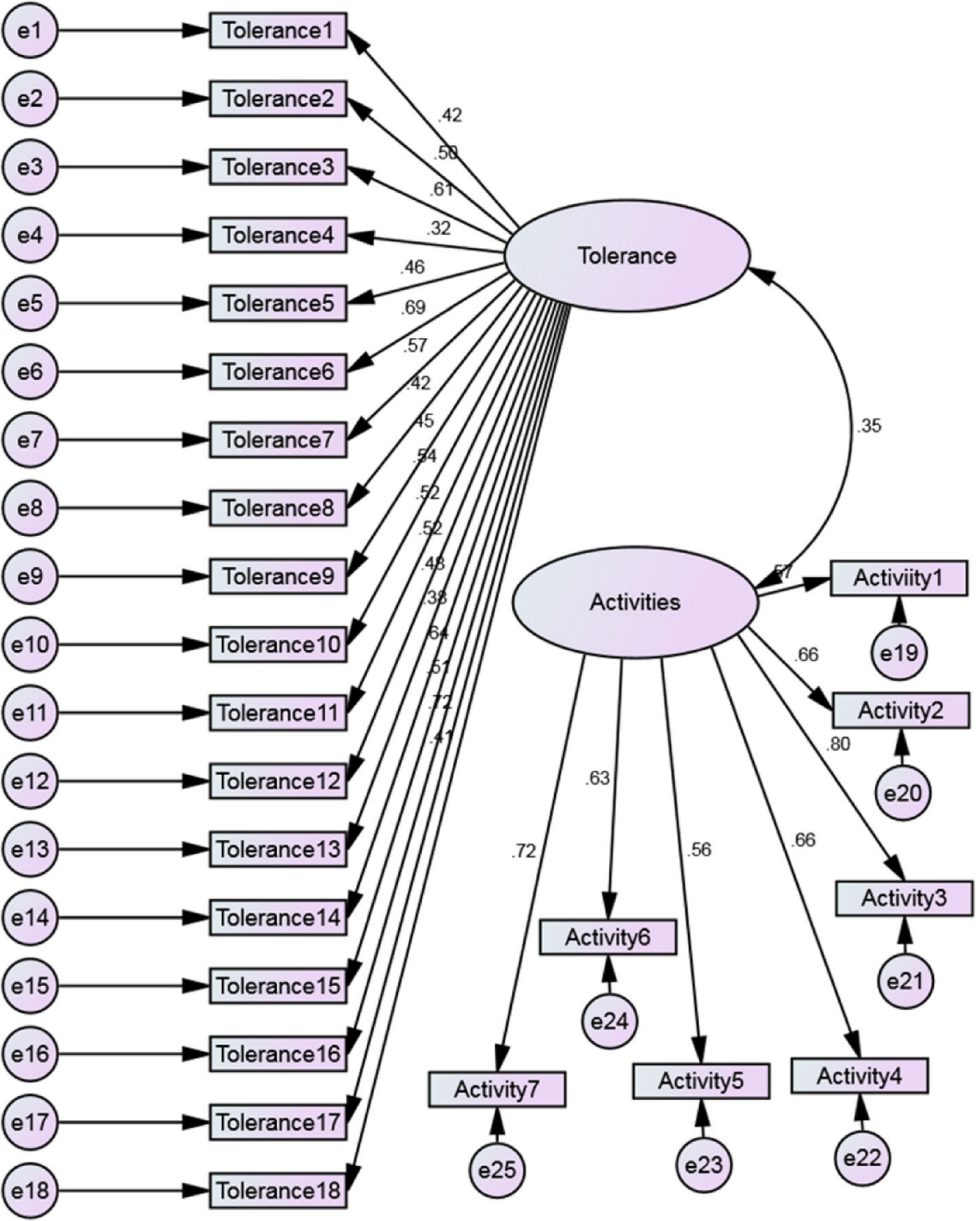
Two-factor structure with maximum likelihood estimation

A confirmatory factor analysis (CFA) was conducted based on a two-factor model as the EFA suggested (Fig. 2). All factor loadings of latent variables displayed in Fig. 2 were above 0.3. A confirmatory factor analysis of the two-factor model showed a good fit, with RMSEA (0.06) ranging between 0.05 and 0.067. Also, CFI and TLI were 0.856 and 0.83, respectively, indicating an acceptable model (Table 4).

**Table 4:** Goodness-of-fit statistics for two-factor model

**Fit Statistic**	**Two-factor model**
Chi^2^	573.461
RMSEA (CI)	0.06 (0.050, 0.067)
CFI	0.856
TLI	0.830

*Note*: *RMSEA* Root mean square error, *CFI* Comparative fit index, *TLI* Tucker-Lewis index, *CI* Confidence interval

## Discussion

In our study, Cronbach’s alpha of the total score was 0.93, which indicates high internal reliability, and the test-retest reliability was 0.79. This excellent value is in concordance with what was found in Ski and colleagues’ (2012) study, in which the Cronbach’s alpha value was 0.87. Ski and colleagues (2012) showed that concurrent validation was good with the Medical Outcomes Study Short-Form 36 (SF-36), BDI and HADS, displaying strong correlations with BDI (r = 0.751, 0.787, 0.737, 0.819) at baseline, 6, 12, and 36 weeks, respectively [33]. The CDS showed 97% sensitivity and 85% specificity for diagnosing major depression [33]. To check the validity of CDS, the SF-36 health survey, BDI and the HADS were all used to explore their correlations with the CDS in the literature. Such a comparison analysis was not performed in this study due to the lack of availability of the Arabic versions of these corresponding scales.

The original scale consisted of 26 items (Cronbach’s α = 0.90) with two robust dimensions and seven subscales [18]. The current study found that the Arabic (Jordanian) version of the CDS presented the same result, showing two dimensions or factors for items of the scale (See Table 3). This result was supported by conducting CFA in the current study. A confirmatory factor analysis was performed using a two factor model. Goodness-of-fit statistics were obtained for the two-factor model and showed that this model showed an overall good fit, with the RMSEA value (0.06) below 0.10, and the CFI (0.856) and TLI (0.83) remaining relatively high, hence indicating acceptable fit (Table 4).

The CDS was translated and validated in an Arabic sample of patients with CVD [24]. While validating the Arabic version of the CDS in Lebanese, the researchers conducted an exploratory factor analysis, revealing three robust factors with a single dimension [24]. Their study reported Cronbach’s alpha of 0.94 for the total scale, and 0.91, 0.86, and 0.87 for the three factors, respectively [24]. These values are close to what was revealed in our study, where Cronbach’s alpha was 0.87 for the total scale, and 0.86 and 0.84 for the two factors, respectively. EFA and CFA in our study suggest two factors for one dimension, which is different from the original validation study of the Arabic CDS that reported three factors [24]. We found one item not loading above 0.3 regarding EFA in the current study, so after removing it, the remaining 25 items of the Arabic (Jordanian) version of CDS captured depression among patients with cardiovascular diseases in Jordan. The item “I wake up in the early hours of the morning and cannot get back to sleep” was removed as it may not assess potential depression among patients with CAD in Jordan. The removed item could be changeable among participants of the current study based on Jordanian culture, and thus prevented the removed item from loading above 0.3. The CFA supported its removal from the instrument, showing acceptable goodness-of-fit indices for a two-factor model with 25 items. It was important to conduct factor analysis for the Arabic (Jordanian) version of CDS since the Jordanian population is a new group, and the Arabic version has never been validated in it.

### Limitations

This study is a secondary data analysis, using data collected from two previous studies, which were cross-sectional studies. Cross-sectional studies do not explore cause-effect relationships. The original studies used self-report questionnaires to measure the main variable of the study, which could be affected by social desirability. This study was unable to address the relationships between depression and other variables, such as healthy lifestyle among patients with CVDs.

## Conclusion

Although the CDS had been translated into the Arabic language previously, differences in Arabic language used in different countries made it essential to translate and validate the Arabic version of the CDS in the Jordanian population to gather accurate data on depression from Jordanian patients with CVD. The Jordanian version of CDS was found to be reliable and valid to assess depression among Jordanian patients with CVD. This instrument might guide a more optimum management of depression. This study provides preliminary information for further comparison research using corresponding validated Arabic depression scales to establish consistent reliability results of the scale overtime in Jordanian population.

## Data Availability

The raw data can be requested from the author: Ibtisam M. Al-Zaru RN, PhD.

## Abbreviations

CVD: Cardiovascular disease
CDS: Cardiac Depression Scale
